# Assessment of Dental Patients’ Awareness of the Correlation Between Systemic and Periodontal Diseases: A Questionnaire-Based Study

**DOI:** 10.7759/cureus.47355

**Published:** 2023-10-19

**Authors:** Nouf Alessa, Wesam Fathi

**Affiliations:** 1 Department of Dentistry, College of Dentistry, Qassim University, Buraydah, SAU; 2 Department of Periodontology, College of Dentistry, Qassim University, Buraydah, SAU

**Keywords:** systemic health, periodontitis, oral health, interrelationship, general public, awareness

## Abstract

Aim: The correlation between periodontal diseases and systemic diseases has been proven. Considering the high prevalence of periodontitis, as well as the high prevalence of systemic diseases such as hypertension, diabetes, and anemia, in Saudi Arabia, patients visiting dental clinics need to be educated about this correlation.

This study aimed to evaluate the knowledge and awareness levels of dental patients on the correlation between periodontal and systemic diseases and compare the awareness levels of the population based on their gender, educational level, and age group specifications.

Methodology: The awareness level of patients was assessed using a questionnaire consisting of 18 questions about the correlation between periodontal and systemic health, along with demographic data (gender, age, and educational level). The questionnaire was distributed to randomly chosen patients who were attending Qassim University dental clinics. Patients could select their responses from three options: “yes,” “no,” and “no idea.” The awareness level of patients was categorized based on their educational qualifications, age, and gender.

Results: A total of 252 patients participated in the study. Out of the 252 patients, 116 (46%) were male, and the remaining 136 (54%) were female. Females were found to be more aware of the correlation between pregnancy and anemia, independently, and periodontal diseases. Additionally, younger age groups were found to be more aware of the interaction between periodontal diseases and hyperparathyroidism. However, the awareness level based on educational qualification was almost equivalent between high school and college graduates.

Conclusion: The general public of the Qassim region of Saudi Arabia needs to be educated further on the correlation between systemic and periodontal disease. Being part of the community, we wish to contribute to improving the level of awareness about the relationship between periodontal and systemic diseases, as our study has concluded that more efforts are required to achieve higher health standards.

## Introduction

Health is a fundamental right and universal human need for all individuals. Without proper oral health, general health cannot be achieved or maintained. Over the last decade, there has been a greater emphasis in dental practice on the importance of oral health as an essential component of overall health and well-being [1].

Furthermore, the correlation between oral health, especially periodontal status, and systemic conditions has been well established [2]. The pathogenesis of periodontal disease was initially only linked to bacterial plaque, but in the last 30 years, our understanding of the pathogenesis of periodontitis has markedly evolved. It is now well known that periodontitis is an infectious disease associated with a small number of predominantly gram-negative microorganisms that exist in a subgingival biofilm [3]. Furthermore, disease initiation and progression are now known to depend on the host’s immune response. Systemic disorders affect the host’s inflammatory mediators by altering the function of the neutrophils, monocytes, macrophages, and lymphocytes, which results in the early onset of periodontal destruction or an increase in the severity of the periodontal disease as compared with cases where such disorders are absent [4]. The adverse effects of periodontal disease on systemic health, such as diabetes, coronary heart disease (CHD), low birth weight at delivery, pre-term labor, and respiratory disease, have been proven [5].

Given the high prevalence of periodontitis in Saudi Arabia and its deleterious association with systemic disease [6,7], patients visiting dental clinics need to be educated on this correlation. Patient education is a strong factor in disease management and prevention as it attempts to change patients’ behaviors by changing their knowledge, beliefs, and attitudes [8].

Previous studies on systemic-periodontal health correlation have been conducted in Saudi Arabia to assess the awareness of dental students, dental practitioners, and medical practitioners [9-11], but there are no studies conducted to assess the awareness of the patients or the general public on this correlation. Therefore, our aim in this study was to evaluate the awareness level of patients attending the Qassim University dental clinics about the correlation between systemic health and periodontal disease. We also aimed to compare the awareness level of patients based on their gender, educational levels, and age group specifications.

## Materials and methods

The study was initiated after obtaining approval from the Dental Ethics Committee of the College of Dentistry, Qassim University (ethical approval code EA/F-2020-5004). The study was conducted in Qassim province. A questionnaire was distributed to randomly selected patients attending Qassim University dental clinics.

Inclusion criteria

A total of 252 patients (136 females and 116 males) attending Qassim University dental clinics who were above the age of 18 and with a minimum qualification of a high school education were included in the study.

Exclusion criteria

Dental students, dentists, and patients who did not meet the minimum educational requirements were excluded.

Method of sampling

A questionnaire containing 18 questions in both Arabic and English languages about the correlation between systemic and periodontal diseases, and demographic data was distributed to randomly selected patients attending Qassim University dental clinics. The questions were classified into eight groups, as shown in Table 1. The patients could choose their answer from three options: “yes,” “no,” and “no idea.”

**Table 1:** Eight categories into which the questions in the questionnaire were divided

Groups	Question’s category
Group A	Question on general awareness toward periodontal diseases and systemic health
Group B	Questions on diabetes and periodontal health relationship
Group C	Questions on pregnancy and periodontal health relationship
Group D	Questions on cardiovascular system diseases and periodontal health relationship
Group E	Question on respiratory system diseases and periodontal health relationship
Group F	Question on anemia and periodontal health relationship
Group G	Question on the effect of hyperparathyroidism on periodontal health
Group H	Question to assess patients’ motivation to maintain good oral health

Statistical analysis

The Mann-Whitney U test was used to measure the association between the variables and determine significant differences between frequencies. The Statistical Package for the Social Sciences (SPSS) version 26.0 (IBM SPSS Statistics, Armonk, NY) was used for the analysis. A p-value of <0.05 was considered significant.

## Results

A total of 252 patients agreed to participate and responded to the questionnaire (Table 2). The approximate time required for a participant to fill out the questionnaire ranged from three to five minutes. Out of 252, 116 (46%) of the sample were males, and 136 (54%) were females. According to the age group specification, 149 individuals were 18-35 years old, 72 individuals were 36-54 years old, and 31 individuals were 55-70 years old. Based on the educational level specification, 84 individuals had completed high school education and 168 were college graduates.

**Table 2:** Demographic data

Demographic data		Total number of participants	Percentage
Gender	Male	116	46%
	Female	136	54%
Age group	18-35 years	149	59.1%
	36-54 years	72	28.6%
	55-70 years	31	12.3%
Educational level	High school	84	33.3%
	College	168	66.7%

**Figure 1:**
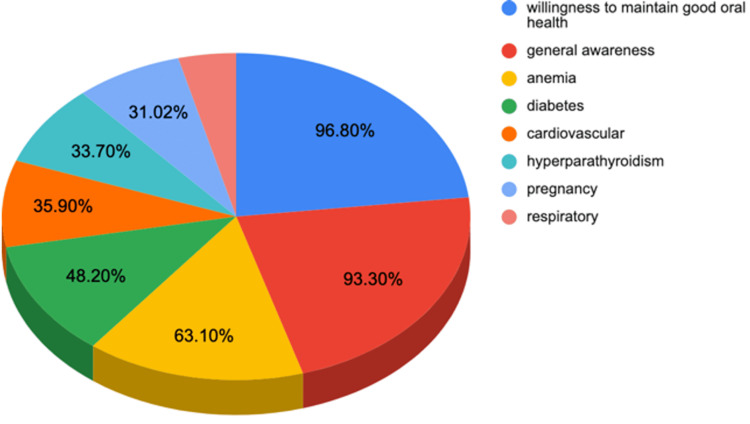
Awareness based on question categories

The overall awareness level of all study subjects about the relationship between periodontal disease and systemic health was 52.3% (Table 3), which is considered average. As shown in Figure 1, the relationship that most individuals were aware of was the one between anemia and periodontal disease, with 63% of the patients being aware of this relationship. Only 48% of the patients were aware of the relationship between diabetes and periodontal health, which is considered low. Patients also displayed low awareness of the relationship between periodontal disease and other systemic diseases related to cardiovascular and respiratory systems, as well as pregnancy.

**Table 3:** Questionnaire with patient responses in percentage

Questions	Percentage		
	Yes	No	No idea
1. Do you think good oral health can lead to improvement in the overall health of an individual?	93.3	2.8	4
2. Do you think that diabetes patients are more prone to gum infection than normal persons?	64.3	8.4	27.4
3. Do you believe that your oral health would be better if you did not have diabetes?	66.7	12.7	20.6
4. Have you ever been told gum disease affects blood glucose control?	26.2	24.6	49.2
5. Are you aware that treatment of gum disease among diabetic patients may help in improving blood glucose?	30.6	15.1	54.4
6. Do you really think there is an association between oral health and diabetes?	53.2	15.1	31.7
7. Do you know that there is an increased tendency for bleeding gums and enlargement of gums during pregnancy?	46	11.9	42.1
8. Are you aware that there is a correlation between oral health and pregnancy outcomes?	31	20.6	48.4
9. Do you think gum disease can cause premature deliveries and low birth weight babies?	12.3	33.7	54
10. Do you think pain in gums or bleeding from gums is normal during pregnancy?	32.5	27.8	39.7
11. Do you think treatment of dental-related problems during pregnancy is safe?	33.3	31.3	35.3
12. Are you aware that microbes causing gum disease can lead to the narrowing of the blood vessels supplying the heart, leading to various heart diseases?	37.3	15.1	47.6
13. Do oral/dental infections affect the heart and other organs?	40.9	18.3	40.9
14. Did you know certain tablets given for blood pressure can increase the size of the gums?	29.4	15.5	55.2
15. Do you think periodontal disease can increase the rate of death in pneumonia patients?	16.7	17.5	65.9
16. Do you think anemia can cause pale gingiva and ulcerations in the mouth?	63.1	6.3	30.6
17. Do you think hyperparathyroidism can affect periodontal health?	33.7	10.3	56
18. If you are told that improving oral health can possibly help you in improving your overall health, would you be more careful in maintaining good oral health?	96.8	3.2	0

The awareness level about the relationship between respiratory diseases and periodontal health was low, with only 17% of the patients being aware of this relationship. When asked whether after being aware of the relationship between periodontal disease and systemic health, they were motivated to maintain good oral health, 96.8% of the patients chose yes.

**Figure 2:**
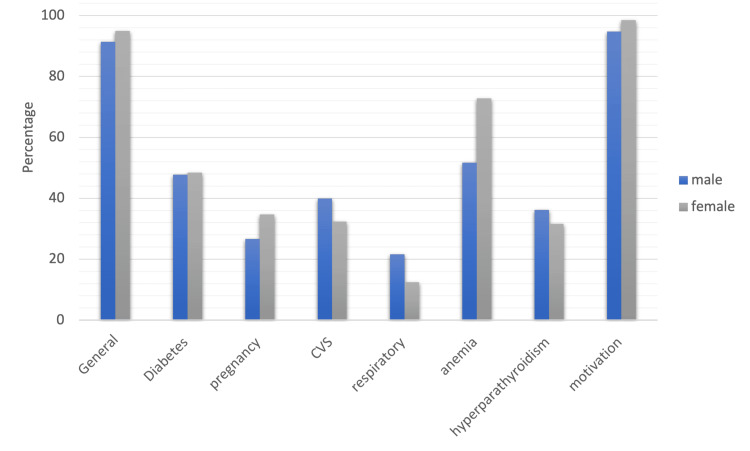
Graph depicting awareness levels based on gender CVS: cardiovascular system diseases

According to gender, awareness levels among male and female groups were nearly the same in all categories except those pertaining to anemia-periodontal health and pregnancy-periodontal health relationships. In these categories, females were more aware, with a significant difference of less than 0.05. Males were more aware of the relationship between respiratory diseases and periodontal health, with a significant difference of less than 0.05, as shown in Figure 2 and Appendices.

**Figure 3:**
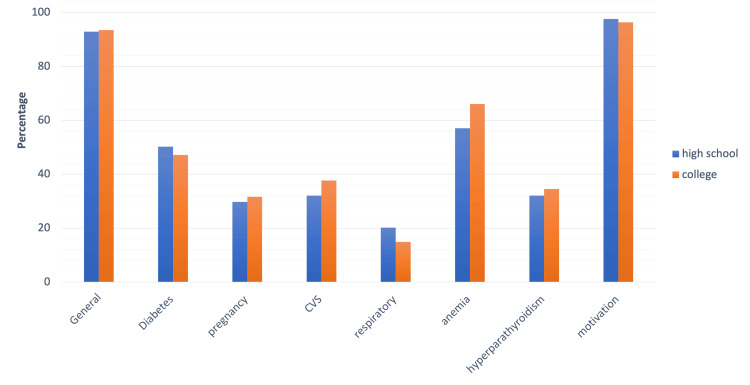
Graph depicting awareness levels across groups based on educational qualifications CVS: cardiovascular system diseases

Based on educational levels, there was no significant difference in the awareness levels about the relationship between periodontal disease and systemic health across the groups (Figure 3 and Appendices), although college graduates were more aware of anemia and CVS relationship with periodontal health.

**Figure 4:**
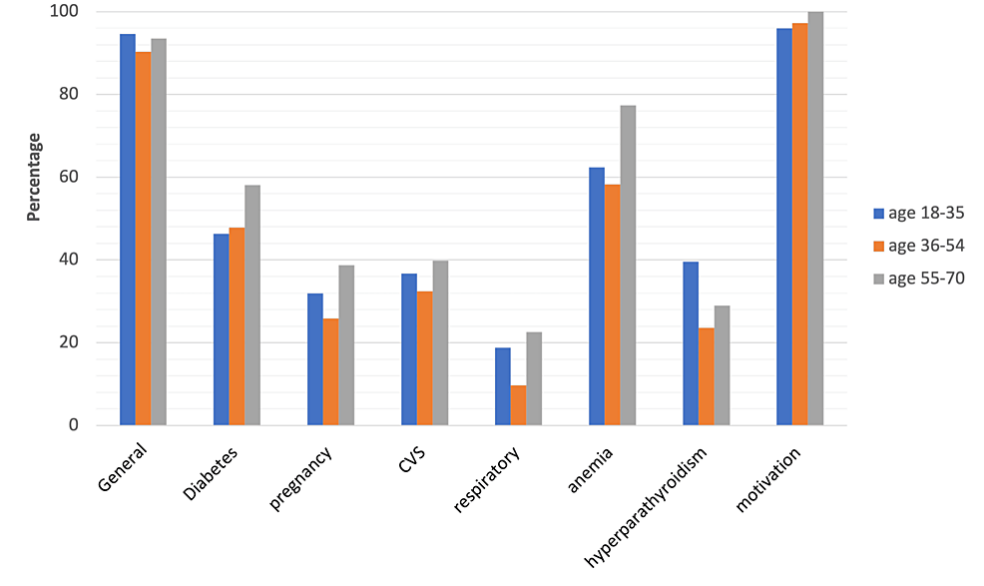
Graph depicting awareness levels across groups based on age ranges CVS: cardiovascular system diseases

Based on age group specifications (Figure 4 and Appendices), there was no significant difference in awareness levels except on the relationship between hyperparathyroidism and periodontal health, where individuals in the age group of 18-36 years were found to be more aware than those in other age groups. Additionally, individuals in the age group of 55-70 years were more aware than the other two age groups about the relationship between pregnancy and periodontal health.

## Discussion

It is well known that periodontal disease, if left untreated, leads to recession, loss of attachment, and mobility, eventually leading to loss of teeth [12]. However, the influence of periodontal disease on systemic health is not as well known as its local effects [13]. This lack of knowledge of the correlation between oral and systemic health often leads to poor systemic health because of poor oral hygiene [14].

The results of our study showed that the awareness level about the overall relationship between periodontal disease and systemic health was 52.3%, which is considered average. This result was found to be contradictory to those from a study conducted by Hemalatha et al. [14], where their results showed a high (78%) awareness level.

In our study, the age group of 55-70 years had the highest awareness level regarding systemic health-periodontal disease correlation, except in the case of hyperparathyroidism and periodontal health, whereas the age group of 18-35 years had the highest awareness level on the subject. This result is considered to be contradictory to those from a study conducted by Gupta et al. [15], where the age group of 35-54 years had the highest awareness in all categories.

Our study also found that the awareness of the female participants was higher than that of the males about the relationship between pregnancy and anemia and periodontal disease, and this result was found to be in agreement with those from the studies conducted by Hemalatha et al. [14], and Singh et al. [16].

Our study found no significant difference between awareness levels based on educational qualifications, which was contradictory to the findings from the studies conducted by Hemalatha et al. [4] and Gupta et al. [15], where the patients with higher educational qualifications showed higher awareness levels.

However, our results concluded that awareness levels were low regarding the correlation between diabetes and periodontal health. A lack of knowledge about this correlation will lead to a lack of patient compliance in the treatment of both periodontal disease and diabetes mellitus. If patients are educated and positively reinforced about the importance of oral health and its association with systemic health, patients will comply with the treatment plan and also make preventive dental visits if they believe they are more susceptible to disease [17].

According to the World Health Organization (WHO), cardiovascular disease (CVD) is the major cause of death globally, contributing to 31% of all mortality cases worldwide [18]. Furthermore, the mortality rate for CVD in Saudi Arabia is even higher, with 42% of all deaths being attributed to CVD [19]. Studies have concluded that periodontal disease is a risk factor for CVD, with a study conducted by Humphrey et al. [20] reporting that patients with different degrees of periodontal disease contribute to a 24%-35% increase in the risk of coronary heart disease (CHD), with the opposite results observed for patients having gingivitis. Based on our results, it was evident that the general public is not aware of the relationship between CVD and periodontal disease. Patients must be educated about the correlation between CVD and periodontal health, especially cardiac patients who might potentially be at a higher risk of cardiac events due to periodontitis.

However, as a limitation of our study, the educational level as well as the age group of our sample was not equally distributed as most of the sample had the same educational qualification and were middle-aged.

## Conclusions

The relationship between periodontal health and systemic health has been established, but general public awareness of this relationship is the most critical aspect in sustaining a healthy community. To summarize, our results concluded that the awareness level of the systemic periodontal correlation in our sample was low. Younger age groups were found to be more aware of the interaction between periodontal diseases and hyperparathyroidism. Females showed higher awareness than males in the correlation between pregnancy and anemia, independently, and periodontal diseases. Population awareness could be raised by community health programs. Collaboration between medical and dental health providers is suggested.

## Appendices

Tables 4-6 present the awareness levels of the participants based on gender specification, educational level, and group specification, respectively.

**Table 4:** Awareness levels based on gender specification p < 0.05 indicates statistically significant results. SD: standard deviation

Question category	Gender	Number	Mean	SD	Mann-Whitney U	p-value
General awareness	Male	116	0.91	0.28	7614.0	0.274
	Female	136	0.95	0.22		
	Total	252				
Diabetes and periodontal health relationship	Male	116	3.30	1.62	7654.5	0.681
	Female	136	3.38	1.61		
	Total	252				
Pregnancy and periodontal health relationship	Male	116	1.34	1.40	6335.0	0.006
	Female	136	1.74	1.26		
	Total	252				
Cardiovascular system diseases and periodontal health relationship	Male	116	1.20	1.1	6942.0	0.084
	Female	136	0.97	1.08		
	Total	252				
Respiratory system diseases and periodontal health	Male	116	0.22	0.41	7174.0	0.055
	Female	136	0.13	0.33		
	Total	252				
Anemia and periodontal health relationship	Male	116	0.52	0.50	6226.0	0.001
	Female	136	0.73	0.45		
	Total	252				
Hyperparathyroidism on periodontal health	Male	116	0.36	0.48	7526.0	0.443
	Female	136	0.32	0.47		
	Total	252				
Motivation to maintain good oral health	Male	116	0.95	0.22	7596.0	0.095
	Female	136	0.99	0.12		
	Total	252				

**Table 5:** Awareness levels based on educational level p < 0.05 indicates statistically significant results. SD: standard deviation

Question category	Educational level	Number	Mean	SD	Mann-Whitney U	p-value
General awareness	High school	84	0.93	0.3	7014.000	0.859
	College	168	0.93	0.2		
	Total	252				
Diabetes and periodontal health relationship	High school	84	3.44	1.7	6768.000	0.591
	College	168	3.29	1.6		
	Total	252				
Pregnancy and periodontal health relationship	High school	84	1.49	1.4	6598.500	0.389
	College	168	1.58	1.3		
	Total	252				
Cardiovascular system diseases and periodontal health relationship	High school	84	0.96	1.1	6367.500	0.184
	College	168	1.13	1.1		
	Total	252				
Respiratory system diseases and periodontal health	High school	84	0.20	0.4	6678.000	0.283
	College	168	0.15	0.3		
	Total	252				
Anemia and periodontal health relationship	High school	84	0.57	0.5	6426.000	0.167
	College	168	0.66	0.5		
	Total	252				
Hyperparathyroidism on periodontal health	High school	84	0.32	0.5	6888.000	0.707
	College	168	0.35	0.47		
	Total	252				
Motivation to maintain good oral health	High school	84	0.98	0.16	6972.000	0.612
	College	168	0.96	0.2		
	Total	252				

**Table 6:** Awareness levels based on age group specification p < 0.05 indicates statistically significant results.

Question category	Age group	Number (%)	Mean	p-value
Pregnancy and periodontal health relationship	18-35	148 (59.1%)	1.59	0.05
	36-54	72 (28.6%)	1.29	
	55-70	31 (12.3%)	1.94	
Hyperparathyroidism and periodontal health relationship	18-35	148 (59.1%)	0.39	0.02
	36-54	72 (28.6%)	0.24	
	55-70	31 (12.3%)	0.29	
